# Remote Bridge Deflection Measurement Using an Advanced Video Deflectometer and Actively Illuminated LED Targets

**DOI:** 10.3390/s16091344

**Published:** 2016-08-23

**Authors:** Long Tian, Bing Pan

**Affiliations:** Institute of Solid Mechanics, Beihang University, Beijing 100191, China; tianl@buaa.edu.cn

**Keywords:** Bridge deflection, video deflectometer, digital image correlation, Wuhan Yangtze river

## Abstract

An advanced video deflectometer using actively illuminated LED targets is proposed for remote, real-time measurement of bridge deflection. The system configuration, fundamental principles, and measuring procedures of the video deflectometer are first described. To address the challenge of remote and accurate deflection measurement of large engineering structures without being affected by ambient light, the novel idea of active imaging, which combines high-brightness monochromatic LED targets with coupled bandpass filter imaging, is introduced. Then, to examine the measurement accuracy of the proposed advanced video deflectometer in outdoor environments, vertical motions of an LED target with precisely-controlled translations were measured and compared with prescribed values. Finally, by tracking six LED targets mounted on the bridge, the developed video deflectometer was applied for field, remote, and multipoint deflection measurement of the Wuhan Yangtze River Bridge, one of the most prestigious and most publicized constructions in China, during its routine safety evaluation tests. Since the proposed video deflectometer using actively illuminated LED targets offers prominent merits of remote, contactless, real-time, and multipoint deflection measurement with strong robustness against ambient light changes, it has great potential in the routine safety evaluation of various bridges and other large-scale engineering structures.

## 1. Introduction

Bridge deflection is an important parameter in safety examination of bridge structures, which reflects the overall stiffness of the bridge structure and is thus closely related to its bearing capacity and the ability to resist dynamic loadings such as traffic, gusts and earthquake. Currently, the most widely used bridge deflection measuring techniques are level gauges and displacement meters, which are relatively accurate, but in general can only be applied to measure static deflection, and thus are not suitable for continuous dynamic measurements. Moreover, these conventional techniques must be operated manually rather than automatically. In addition, these contacting measurement methods require the sensors be attached to a test bridge’s main beams, and some displacement sensors require fixed reference benchmarks as an installation platform. Considering that many bridges are built over highways, water, mountains and valleys, it is difficult to install and uninstall reference benchmarks and displacement sensors, which makes some important parts hard to be reached and tested and, thus, greatly limits the applications of these contact sensors.

To overcome the limitations of traditional contract sensors, various non-contact displacement measurement systems using GPS technology [1,2], laser Doppler vibrometry [3], radar interferometry [4], robotic total station [5], and vision-based (or image-based) optical techniques [6,7,8,9,10,11,12,13,14], have been developed and advocated for bridge deflection measurement with their claimed advantages. It is also noted that some researchers combined several of these technologies [2,15,16] to extend the application range and improve the sensitivity and accuracy of the measurement system. Despite the diversity of non-contact deflection measurement techniques, vision-based optical techniques have received increasing attention due to their outstanding advantages, such as low-cost and easy-to-use setup, multipoint real-time measurement and visualization, and wide range of resolution and applicability.

Recently, a self-developed advanced video deflectometer based on the principle of off-axis digital image correlation (off-axis DIC) was developed by the present authors for remote, real-time, multipoint, and targetless bridge deflection measurement [14]. The video deflectometer relies on the natural texture on the bridge to track the vertical motion (i.e., deflection) of test bridges. With the aid of the state-of-the-art subpixel registration algorithm, the video deflectometer is able to provide real-time and high-accuracy deflection measurement provided that the texture on the test bridge has sufficient and constant intensity contrast during the whole measurement period. However, in certain cases, especially for deflection measurement of very large bridges at remote distance or at night, where sufficient and constant illumination is difficult or impossible, the texture of the bridge tends to be indiscernible, thus leading to a failure of targetless deflection measurement using the video deflectometer. In particular, to avoid the interference of daily traffic and to ensure the measurement accuracy, many safety examinations of bridges have to be carried out at night when these bridge are closed to traffic. During the night, one of the major challenge is that the visibility and resolution of the natural textures on the bridge are insufficient to be tracked by even the most robust image registration algorithms.

To address the challenge of remote and accurate deflection measurement of large bridges at night, the novel idea of active imaging combining high-brightness monochromatic LED targets and bandpass filter imaging, which was originally proposed in [17,18] to realize deformation measurement in outdoor or high-temperature environments, is introduced. Thanks to the easy-to-implement but robust active imaging technique, the ambient light from the road lamps on the bridge can be effective suppressed to ensure high-accuracy motion tracking of LED targets. Compared with traditional contacting displacement measurement techniques, the video deflectometer has the advantages of compact configuration, ease-of-use, as well as remote, real-time, and multipoint deflection measurement. Additionally, in contrast to other vision-based optical techniques using various targets [6,7,8,9,10,11,12,13,14], the proposed technique offers outstanding advantage of being insensitive to ambient light variations just with a simple improvement made on LED targets and the optical imaging system.

In the rest of this paper, system configuration, basic principles and measuring procedures of the video deflectometer are briefly described. Then, the accuracy of the video deflectometer in outdoor environments was characterized using in-plane vertical translation tests performed at night. Finally, field deflection measurement of the Wuhan Yangtze River Bridge with the aid of high-brightness red LED targets is demonstrated.

## 2. An Advanced Video Deflectometer for Real-Time, Remote Bridge Deflection Measurement

### 2.1. System Configuration

**Figure 1:**
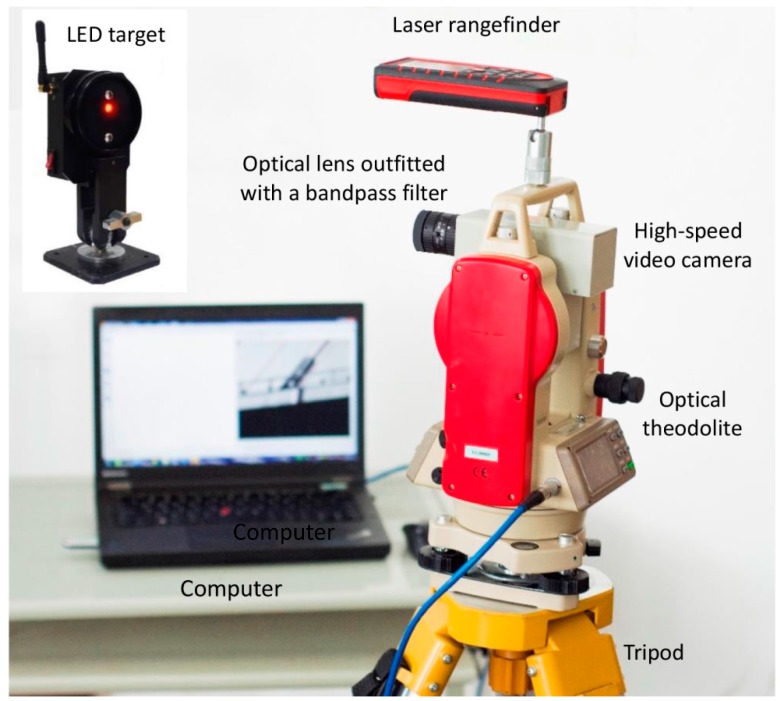
The established video deflectometer for bridge deflection measurement.

Shown in Figure 1 is the self-developed video deflectometer for real-time, remote, and multipoint bridge deflection measurement, which consisted of a high-speed area scan monochrome camera (Genie HM1024, Teledyne DALSA, Waterloo, ON, Canada, resolution: 1024 × 768 pixels with 8-bit quantization, maximum frame rate: 117 fps), a fixed-focal optical lens (the focus length of the lens can be changed as per actual measurement requirements), a laser rangefinder (BOSCH GLM 250 VF Pro, Robert Bosch GmbH, Power Tools Division, Baden-Württemberg, Germany, measuring range: up to 250 m, accuracy: 1 mm), an optical theodolite, and a laptop computer (Thinkpad T440p, Lenovo, Beijing, China, Intel(R) Core(TM) i7-4700MQ CPU, 2.40 GHz main frequency and 8 GB RAM). The video camera is fixed onto a platform that is attached onto the horizontal axis of the optical theodolite. By use of the optical theodolite, the pitch angle and horizontal (or yaw) angle of the video camera can be adjusted readily and then fixed tightly. The camera equipped with an optical imaging lens is connected to the laptop computer using a gigabit Ethernet standard LAN wire, through which the live video images of a test bridge can be displayed in real-time. These live video images can be processed by the state-of-the-art DIC algorithm [14,19] described later to extract image motions (in pixels) at specified measurement points, which can be subsequently converted into desired physical displacements (deflections) in millimeters based on an easy-to-implement, but accurate, calibration model introduced in the following section.

It is quite necessary to note that, as an intensity-based image registration algorithm, DIC relies on the random intensity distribution on the test object to realize accurate subpixel displacement tracking. The developed video deflectometer can operate with two different kinds of measurement modes, namely targetless mode and target mode. The targetless measurement mode takes advantage of the natural texture (e.g., traffic sign, various varying structures) on a test bridge or structure, as evidenced in our recent work [14]. The target measurement mode utilizes artificial targets (typically, specially-designed high-brightness LED lamps) attached onto the test bridge, which facilitates the displacement tracking or deflection measurement of textureless surfaces or during the night.

It is important to note that, to effectively suppress ambient light variations in outdoor environments and/or during the night and ensure high-accuracy target tracking, the simple, but robust, active imaging technique, which was originally proposed by Pan et al. [17,18] to realize deformation measurement in outdoor or high-temperature environments, was incorporated into the established video deflectometer. To be specific, high-brightness red LED lamps emitting at a wavelength of 620 ± 3 nm with a power of 3 W were used as actively-illuminated targets. Additionally, a coupled bandpass optical filter with a center wavelength of 620 nm and a full-width at a half-maximum value of 30 nm, which merely allows the red light to pass through, is mounted before the lens of the video deflectometer to minimize the adverse influence of ambient light variations. Since the random noise in the video images due to ambient light can be greatly suppressed, the accuracy and precision in displacement tracking are expected to be improved by using actively-illuminated LED targets. In short, compared with existing video-based systems [6,7,8,9,10,11,12,13,14] for bridge deflection monitoring, which mainly relies on precise targets with evident geometric characteristics and customized image processing algorithms, the present video deflectometer is more practical, robust and versatile.

### 2.2. Measurement Procedures and Principles

The bridge deflection measurement using the established system involves the following three steps:

(1) Measurement Preparation

Place the video deflectometer on the ground that permits imaging the test bridge far away. Note that the measuring system should be kept stationary during the whole measurement period, because small vibrations of the camera can lead to considerable displacement errors [12,20]. Then, tune the imaging distance and aperture of the lens to get a clear image of the bridge with sufficient contrast and without overexposure. Afterwards, specify discrete measurement points in the live image of the test bridge. For each measurement point a subset with proper size should be chosen, which should contain sufficient local natural texture or a pre-installed LED target to allow an accurate subset-based pattern matching. In general, the optical axis of the video camera is oblique to the test bridge surface, which implies the magnification factor for each measurement point is different. To calibrate the corresponding magnification factor for each point, the distance *D* from the camera sensor to each measurement point is measured using the laser rangefinder. Thus, the objective distance of the point (i.e., the distance from optical center to the measurement point) *L* can be estimated as *L* = *D* − *f* with *f* being the focus length of the camera lens. Additionally, the pitch angle *β* of the camera can either be measured by the laser rangefinder or by the optical theodolite. These two parameters will be subsequently used to convert image displacements to real displacements using the calibration model described below.

(2) Displacement Tracking Using DIC

After defining the measurement points and corresponding subsets, the image displacements of these interrogated points can be tracked by an efficient but accurate inverse-compositional Gauss-Newton (IC-GN) algorithm. More details of the subset tracking using the IC-GN algorithm can be found in our previous publications [14,19], and is briefly reviewed hereafter. To be specific, the displacements at each measurement point can be computed with subpixel accuracy by optimizing the by optimizing the following robust zero-mean normalized sum of squared difference (ZNSSD) criterion.

(1) $$C_{\text{ZNSSD}}(\Delta p)=\sum_{\xi }{\left\{\frac{\left[f\left(x+W\left(\xi ;\Delta p\right)\right)-\bar{f}\right]}{\Delta f}-\frac{\left[g\left(x+W\left(\xi ;p\right)\right)-\bar{g}\right]}{\Delta g}\right\}}^{2}$$

where *f*(***x***) and *g*(***x***) denote the grayscale levels at ***x*** = (*x*, *y*)^T^ of reference image and the deformed image, $\bar{f}=\frac{1}{N}\sum_{\xi }f\left(x+W\left(\xi ;\Delta p\right)\right)$, $\bar{g}=\frac{1}{N}\sum_{\xi }g\left(x+W\left(\xi ;p\right)\right)$ are the mean intensity value of the two subsets, $\Delta f=\sqrt{{\sum_{\xi }\left[f\left(x+W\left(\xi ;\Delta p\right)\right)-\bar{f}\right]}^{2}}$ and $\Delta g=\sqrt{{\sum_{\xi }\left[g\left(x+W\left(\xi ;p\right)\right)-\bar{g}\right]}^{2}}$. $\xi ={\left(\Delta x,\Delta y\right)}^{T}$ is the local coordinates of the pixel point in each subset. W(ξ;p) is the warp function, also known as the displacement mapping function (or shape function) in DIC, depicting the position and shape of the target subset relative to the reference subset; W(ξ;Δp) is the incremental warp function exerted on the reference subset.

Despite that various (e.g., zero-, first-, and second-order) shape functions can be used in the IC-GN algorithm to approximate the possible local deformation of the target subsets, the use of overmatched higher-order shape functions with heavier computational complexity has not shown an improvement in displacement measurement accuracy for remote bridge deflection measurement [21]. Due to this reason, only the simplest, but practical, zero-order displacement mapping function, which only allows rigid body translation of the target subset, is used. Thus, the ZNSSD criterion becomes a non-linear objective function with respect to two displacement components, which can be solved iteratively using the IC-GN algorithm and bicubic spline interpolation. Benefiting from the high-efficiency and high-accuracy IC-GN algorithm, the video deflectometer can realize real-time displacement tracking at 30 discrete measurement points at a frame rate of 117 fps [14].

(3) Converting Image Displacement to Physical Displacement

**Figure 2:**
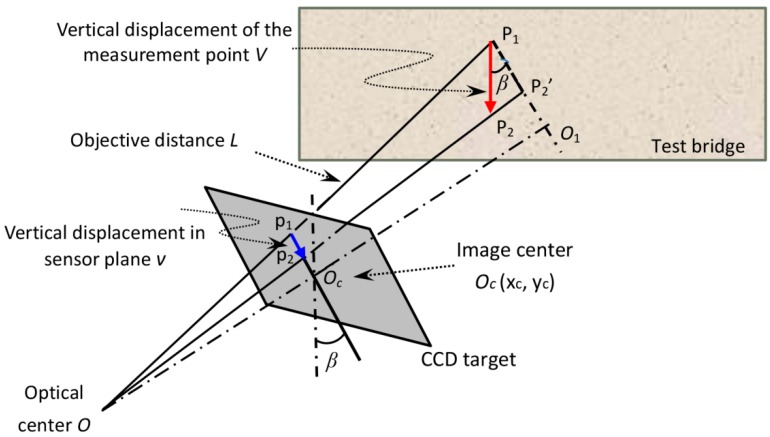
Schematic representation of the calibration model.

In field measurement of bridge deflection using the proposed video deflectometer, the optical axis of the video camera is no longer perpendicular to the test surface of the test bridge. Consequently, the pitch angle and yaw angle of the camera are no longer zero values. However, since the established video deflectometer was designed to measure deflection (i.e., vertical displacement), the yaw angle has no effect on the measured vertical displacement and, thus, is not indicated in the calibration model (Figure 2). In this case, the magnification factor at each measurement point is not a constant, but depends on various parameters including the image coordinates of the measurement point (*x*, *y*), the pitch angle *β* of the camera, the focus length *f* of the camera lens, as well as the object distance *L* of the measurement point. To determine the position-dependent magnification factor for video deflectometer, the pinhole camera model is used to relate an object point with its corresponding image point, as schematically shown in Figure 2. Assume a measurement point *P*_1_ on the test bridge undergoes a vertical motion *V* and moves to *P*_2_. Thus the corresponding image point moves from *p*_1_ to *p*_2_ on the CCD sensor target. To facilitate understanding, an auxiliary line *P*_1_*O*_1_//*p*_1_*O_c_* is plotted in Figure 2. Thus, the extension of *OP*_2_ intersects *P*_1_*O*_1_ at point *P*_2_′. According to homothetic triangle theory, it can be seen from Figure 2 that:

(2) $$\frac{P_{1}{P_{2}}^{\prime }}{p_{1}p_{2}}=\frac{OP_{1}}{Op_{1}}=\frac{L}{\sqrt{\left[{\left(x-x_{c}\right)}^{2}+{\left(y-y_{c}\right)}^{2}\right]l_{ps}^{2}+f^{2}}}$$

where (*x*, *y*) and (*x*_c_, *y*_c_) are the image coordinate of the measurement point and the optical center, respectively. Note that the latter is assumed to be located in the image centre for simplicity; $l_{ps}$ = 7.4 μm is the physical size of each pixel unit; *f* is the focal length of the camera lens used in the following tests. *L* is the distance from optical center to the measurement point, which can be measured using the laser rangefinder.

In general, the focal length of the camera lens is much larger than the half height of the sensor target (=2.84 mm), thus the angle ∠*OP*_2_′ *P*_1_ approximates to 90°, and the desired vertical displacement of the interrogated point can be computed as:

(3) $$V≅\frac{L}{\sqrt{\left[{\left(x-x_{c}\right)}^{2}+{\left(y-y_{c}\right)}^{2}\right]l_{ps}^{2}+f^{2}}}\frac{vl_{ps}}{\cos\beta }$$

where the pitch angle *β* of the camera can also be measured either by the laser rangefinder or the optical theodolite.

## 3. Accuracy Validation of the Video Deflectometer for Tracking LED Targets

**Figure 3:**
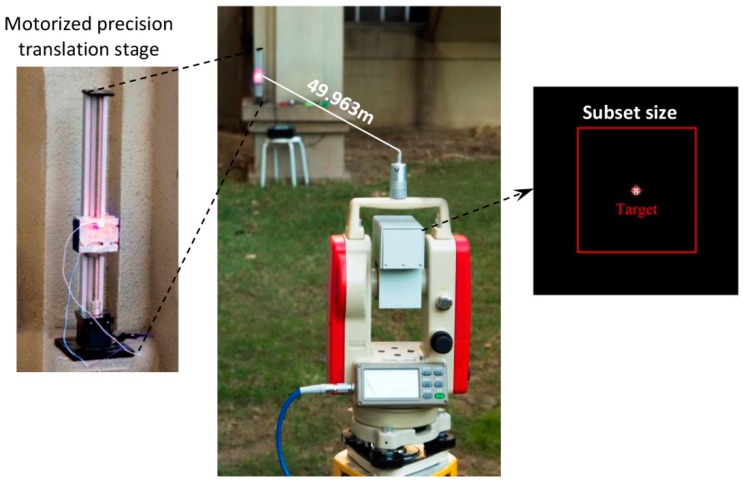
Tracking the movements of an LED lamp using the video deflectometer: the left insert shows the motorized precision translation stage and the right insert indicates an enlarged video image of the LED target.

To validate the measurement accuracy of the established video deflectometer in outdoor environments during the night time, a group of in-plane translation tests along the vertical direction were carried out using a motorized precision translation stage equipped with a red LED lamp as shown in Figure 3. The red LED lamp emitting at a wavelength of 620 ± 3 nm with a power of 3 W was attached onto the sliding block of the translation stage, which is subsequently used as the target point tracked by the proposed advanced video deflectometer outfitted with a coupled bandpass optical filter.

In the following translation tests, the video deflectometer using an optical lens with a fixed focus length of 50 mm was placed 10.605 m, 49.963 m, 101.428 m, 201.485 m, and 300.152 m, respectively, away from the motorized precision translation stage. Then, based on these distances measured by the laser rangefinder and the pitch angle indicated on the optical theodolite, which was set to be 0 degrees in these tests, the magnification factors at different distances can be precisely calculated according to Equation (3). It should be noted that, in the first two translation tests, the sliding block moved upwards by 60 mm at a speed of 50 mm/s, and the sliding block paused for two seconds every 2 mm, whereas the sliding block was reset to pause for 10 s every 10 mm translation in the later three tests.

**Figure 4:**
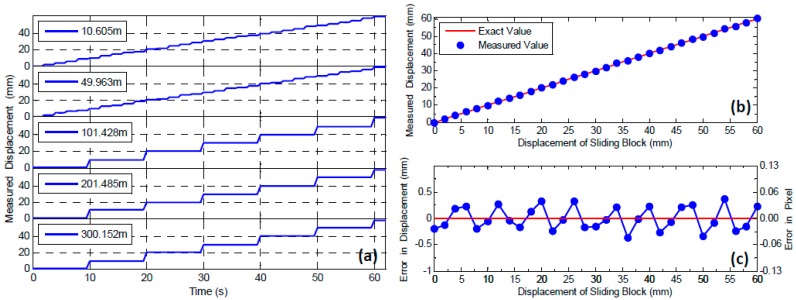
(**a**) Measured vertical motions of the LED target at different distances; (**b**) comparison of the measured results to the actual values at the distance of 49.963 m; and (**c**) the differences between the measured results and the actual value at the distance of 49.963 m.

The vertical translations of the LED lamp were tracked by the video deflectometer in real time. The left part of Figure 3 shows a real image of the LED lamp recorded by the video deflectometer placed 49.963 m away. It is evident that ambient light has been successfully suppressed thanks to the attached bandpass optical filter. To accurately track the position of the LED lamp, a subset of 41 × 41 pixels centered at the LED lamp was chosen. The detected vertical displacements for all the five translation tests are plotted as a function of time in Figure 4a, in which the ladder-like displacement curves are in perfect agreement with preset motions.

To quantitatively examine the accuracy of the video deflectometer, the average displacement at each dwell time was estimated. A comparison between the averaged measured displacements of the red LED lamp (indicated by blue circle dots) with the applied ones (solid line) for the third translation test with a distance of 49.963 m is shown in Figure 4b. The differences between the measured results and the actual values are plotted in Figure 4c, in which the left label shows the errors in pixels. It is observed that these data points distribute averagely on both sides of the zero line, with an estimated maximum error of 0.42 mm (corresponds to 0.0508 pixels). The approximately sinusoidal distributed error curve can be interpreted as the systematic errors associated with image noise and imperfect subpixel interpolation algorithm [22,23,24]. It should be emphasized here that the interference of ambient vibrations or possible winds in outdoor environment was not considered in this work which, generally, greatly lessened the measurement accuracy of the established video deflectometer according to our experiences.

Table 1 summarizes the mean errors and standard deviation errors of vertical displacements measured by the video deflectometer at different measuring distances. It is observed that the mean error and standard deviation error increase almost exponentially with the increases of measuring distance. The mean error was estimated as 0.5674 mm for the maximum measuring distance of 300.152 m, which is comparable to that evaluated from the deflection curve of the real bridge test before loading and after loading. The rapid non-linear increase of measurement errors can be explained by the following two aspects: (1) with the increase of measuring distances, the corresponding image size of the LED target will decrease as a function of the square of the measuring distance. As a result, valid grayscale information used for target tracking, which can be characterized by a parameter called the sum of square of subset intensity gradient (SSSIG) [25], is decreased greatly; and (2) the influences from winds, lights, and ambient vibrations in an outdoor environment are more pronounced at longer measuring distances. It should be noted that we have conducted the same translation tests in the daytime. Although the ambient light mainly due to sunlight can also be effectively suppressed, we observed that the errors in measured displacement are almost two times larger than those measured during the night. The enhanced accuracy of the video deflectometer using actively-illuminated LED targets at night is due to the fact that the ambient light during the night time is much weaker than sunlight and, thus, has less contribution to the image noise.

## 4. Deflection Measurements of the Wuhan Yangtze River Bridge under Static Loading

The Wuhan Yangtze River Bridge [26], with a length of more than 1670 m, was opened to traffic in 1957. The upper layer of the bridge is highway, and the nether layer is double-track railway. There are eight piers and nine apertures, and the distance between each two piers is 128 m, under which ships as heavy as tens of thousands tons could pass through. For every three apertures, there is a continuous beam, and the whole bridge is constituted of three continuous beams. As one of the most prestigious and most publicized constructions in China, the Wuhan Yangtze River Bridge is not only the first double-track railway and highway bridge built across the Yangtze River since the founding of the People’s Republic of China, but also the first Yangtze river bridge, and is generally referred to as *the First Bridge of the Thousands of Miles Yangtze River.* The bridge links the three towns in Wuhan city, promoting traffic convenience and economic development tremendously. Meanwhile, the bridge connects South China and North China, and the Beijing–Hankou railway and the Canton-Hankou railway are also linked by the bridge as the complete Beijing-Guangzhou railway, playing an important role in promoting the north-south economy and constructing the national economy.

Due to the fast economic development of China, the 58-year-old Wuhan Yangtze River Bridge is facing increasingly heavier missions than when it was designed. At present, 296 trains pass through the bridge in a day, approximately a train every five minutes, on average. Meanwhile, passing motor vehicles have escalated from thousands at the beginning to more than a hundred thousand. To examine the real technical status of the bridge structure, and ensuring its safety and reliability, the Wuhan Railway Bureau conducted a fully safety examination from 3 to 9 June 2015. The examination includes static loading and dynamic loading tests, expecting to fully evaluate the working performance of the bridge structure in different conditions, so as to provide a scientific basis for the bridge maintenance.

**Figure 5:**
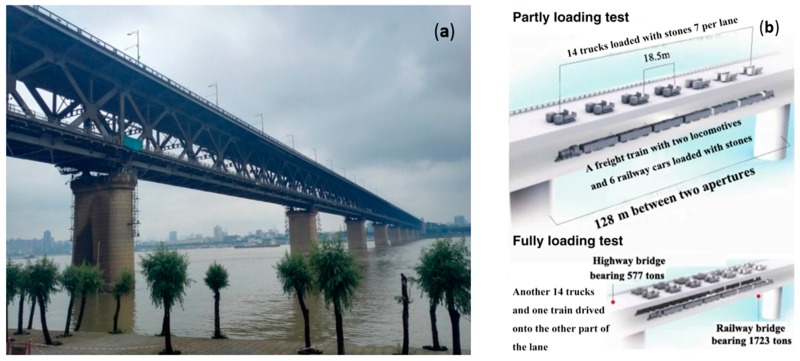
(**a**) Wuhan Yangtze River Bridge; and (**b**) the schematic of the static loading test.

In the examination, the bridge’s second aperture was loaded by vehicles, while deflections of the second and third apertures were measured by the video deflectometer. Figure 5a shows a real picture of the bridge and a schematic figure of the loading cases. Vehicles on the highway and trains on the railway were loaded simultaneously and stopped at the same place as schematically shown in Figure 5b. Two motorcades and two trains were used in the examination. Each motorcade had six trucks whose full load is 180 KN (18.37 tons) and a truck whose full load is 300 KN (30.61 tons). The trains were pulled by two HXD1B locomotives at the head and the end, with six fully-loaded C70 railway cars in between, weighing 861.6 tons. Details of the three loading cases are given as follows:

(1) ***Partly loading on the upward way*:** the vehicles and the trains were loaded on the upward way simultaneously;
(2) ***Full loading*:** the vehicles and trains were loaded on both the upward and downward ways;
(3) ***Partly loading on the downward way*:** unload the vehicles and cars on the upward way and remain the loads on the downward way.

Finally, the loads exerted on the downward way were removed, and the examination was ended.

**Figure 6:**
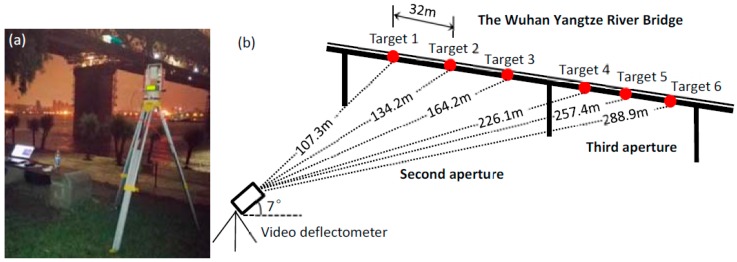
(**a**) The video deflectmeter placed at the riverside; and (**b**) the distances and orentation of the video deflectometer relative to the six measuring points.

To avoid the interference of daily traffic and ensure the measurement accuracy of static deflection, the examination was executed with the bridge was closed to traffic at night. Since the visibility and the resolution of the bridge are insufficient to be discerned, six high-brightness red LED targets were installed equally on the second and third apertures. The horizontal distance between adjacent two LED targets is 32 m, as shows on Figure 6b. The video deflectometer was placed at the riverside (Figure 6a). By using an optical lens with a fixed focus length of 8 mm, and adjusting the orientation of the video deflectometer, all six LED targets can be discerned in images without overlap. Then, the distances from the camera sensor to the targets, as well as the pitch angle of the camera, were measured using the rangefinder. In this examination, the distances from the camera sensor to the six LED targets are measured as 107.3 m, 134.2 m, 164.2 m, 226.1 m, 257.4 m, and 288.9 m, respectively, and the pitch angle is determined as 7 degrees. Note that the distances from the latter three LED targets to the video deflectometer are larger than the maximum measuring distance of the laser rangefinder. In reality, these distances were not measured but estimated from geometric relationships between these regularly-spaced targets. All of these parameters are subsequently input to the video deflectometer software to calculate the magnification factor for each measurement point.

**Figure 7:**
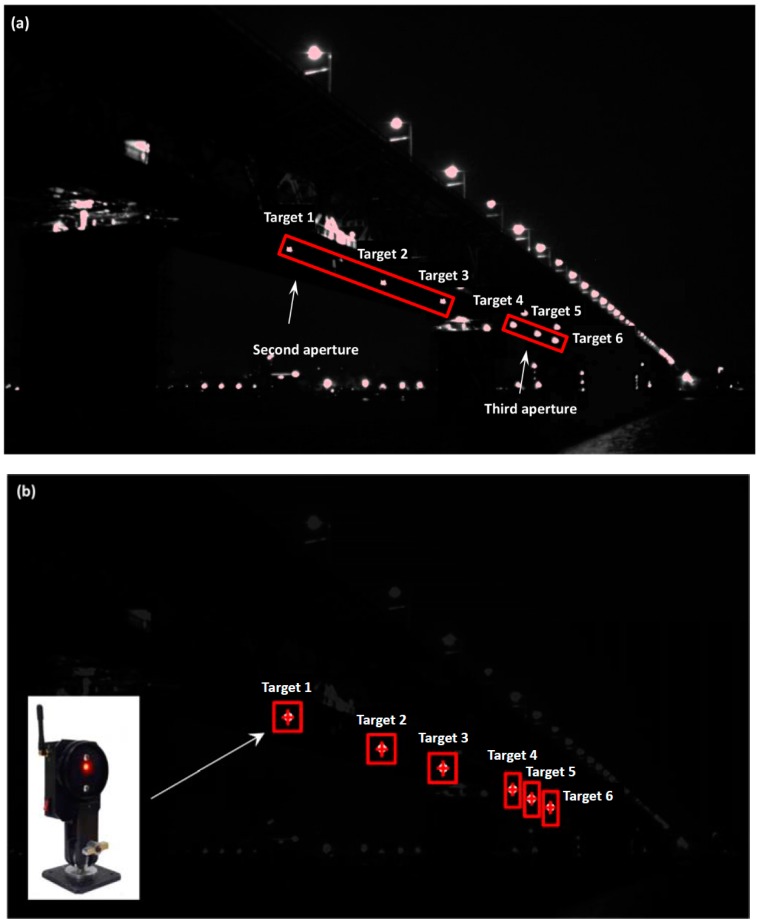
The digital images of the Wuhan Yangtze River bridge (**a**) without; and (**b**) with using the bandpass optical filter. The red rectangles are the subsets centered at the six LED targets.

Figure 7a,b show real images of the Wuhan Yangtze River bridge without and with using the bandpass optical filter. In contrast to Figure 7a, it is seen that the ambient light mainly from road lamps was almost completely suppressed in Figure 7b due to the use of the bandpass optical filter. Then, to determine the image motions of the six LED target, six rectangular subsets centered at these targets were selected. Note that for the left three targets, two square subsets with a size of 41 × 41 pixels were chosen. However, to exclude the interference of other targets, rectangular subsets were chosen for the other four targets.

**Figure 8:**
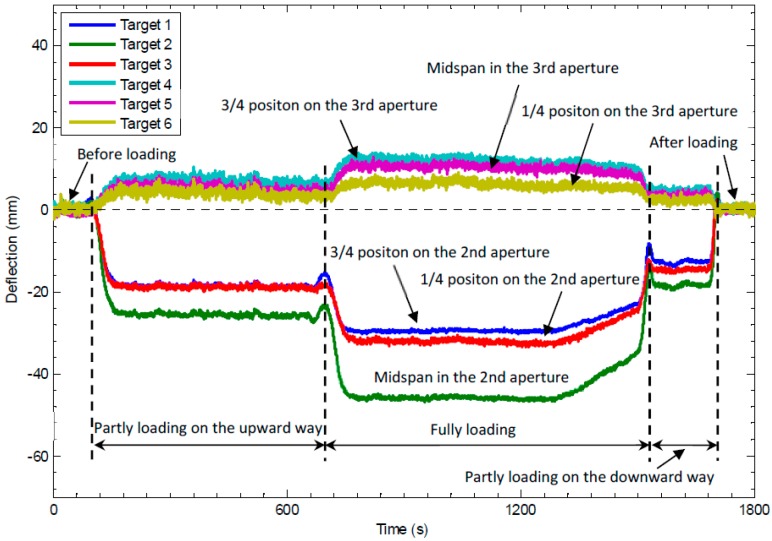
Measured deflection-time curves for the six targets.

Figure 8 shows the deflection-time curve of the six measurement points evenly located on the second and third apertures. It can be observed that: (1) before loading and after loading, the deflections of all the six measurement points fluctuate around zero with an estimated standard deviation ranging from 0.19 mm to 0.57 mm, demonstrating the good repeatability of the video deflectometer; (2) the deflection directions of the three points on the second aperture and the three points on the third aperture are opposite, which is consistent with actual deformation case of the bridge. The second aperture sank due to the applied loading, which the cocked third aperture can be well explained by the continuity of the beam; (3) on the second aperture, the deflection of the point in the middle is always larger than the other two, which are approximately the same because of symmetry; and (4) on the third aperture which is cocking, the point at the 1/4 position is larger than the point at the middle and the point at the 3/4 position. This can be also explained by the deformation continuity of the continuous beam. Also, the maximum deflection of the middle point on the second aperture under full loading was measured to be 47.4 mm. 

To quantitatively evaluate the accuracy and precision of the video deflectometer for field measurements, the measured deflection curves of the six LED targets, lasting 80 s before loading, are further analyzed to extract their mean error and standard deviation error. In this period, the actual deflections of the six LED targets should be zeros. Thus, the estimated errors in the detected deflections also reflect the accuracy and precision of the video deflectometer for different measuring distances. As shown in Table 2, although the mean errors randomly fluctuate around zero values, the standard deviation errors increase with the increase of the measuring distances. Further, these observed standard deviation errors are almost at the same magnitude as those measured from the validation tests. The small differences in standard deviation errors between field measurements and validation tests are acceptable, considering the optical lens used and the measuring environment of these two tests are distinctly different.

**Figure 9:**
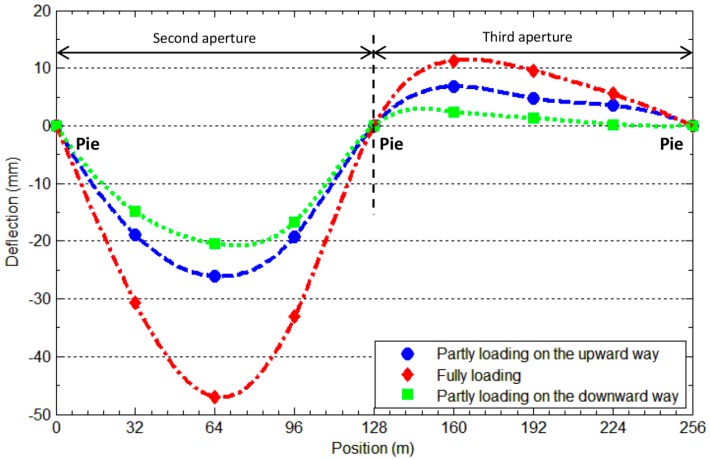
Measured deflection curves for the two spans under three different loading conditions.

To intuitively show the overall deformation of the bridge, the deflection curves of the six points on the second and third apertures in this static loading examination are shown in Figure 9. It can be seen more clearly that the second aperture sank and the third aperture cocked in all of the three loading cases. Moreover, as for the case of partial loading on the downward way, the load is applied to the place relatively far away from the six measurement points. Consequently, the resulting deflections are larger than those measured for partial loading on the upward way, in which the same load was exerted on the position closer to these target points. These different deformations are also reasonably shown in Figure 9.

## 5. Conclusions

Real-time, remote, and field deflection measurement of the Wuhan Yangtze River Bridge at night using an advanced video deflectometer and actively illuminated LED targets is described in this work. The basic principles and measuring procedures of the video deflectometer are briefly reviewed. The accuracy of the video deflectometer in the similar conditions (outdoor environments during the night time) was carefully characterized by detecting vertical motions of an LED target with precisely controlled translations. The deflection-time curves of the six measurement points evenly located on the two apertures of the bridge were measured, and the overall deflection curves at different loading cases were presented. All of the measured deflections agree well with practical loading conditions. Since the proposed video deflectometer offers prominent advantages of remote, contactless, robust, real-time, and multipoint deflection measurement at both targetless and target modes, it demonstrates great potential in the routine safety evaluation of various bridges and other large-scale engineering structures.

## Figures and Tables

**Table 1:** Measurement errors of the video deflectometer at different measuring distances.

Measuring Distance (m)	Mean Error (mm)	Standard Deviation (mm)
10.605	0.0091	0.0074
49.963	0.0150	0.0695
101.428	0.0970	0.2021
201.485	−0.1542	0.2795
300.152	0.5674	0.5897

**Table 2:** Mean errors and standard deviation errors of the deflections measured for the six LED targets in the stationary condition.

Target No.	Measuring Distance (m)	Mean Error (mm)	Standard Deviation (mm)
1	107.3	−0.1566	0.1960
2	134.2	0.1898	0.2337
3	164.2	0.1190	0.2978
4	226.1	−0.1157	0.4783
5	257.4	0.2107	0.4519
6	288.9	0.0204	0.5712
